# Naturwissenschaftliche Grundlagen der Apothekerausbildung

**DOI:** 10.1007/s00103-025-04035-3

**Published:** 2025-04-02

**Authors:** Bernd Clement

**Affiliations:** 1 Konferenz der Fachbereiche Pharmazie, Kiel, Schleswig-Holstein Deutschland; 2 Pharmazeutisches Institut der Universität Kiel, Gutenbergstraße 76, 24118 Kiel, Deutschland

**Keywords:** Pharmazie, Naturwissenschaften, Ausbildung, Approbation, Grundstudium, Pharmacy, Natural sciences, Education and training, License to practice, Basic studies

## Abstract

Pharmazie ist Bestandteil der naturwissenschaftlichen Fächer und bildet dementsprechend auch auf Basis der Naturwissenschaften aus, wobei im Gegensatz zu den anderen naturwissenschaftlichen Fächern die Pharmazie durch eine bundeseinheitliche Approbationsordnung geregelt ist. Dabei sind besonders allgemeine, organische und physikalische Chemie, Analytik, Biologie, Biochemie, Humanbiologie, Mathematik und Physik erwartungsgemäß als Grundlagenfächer vorgesehen. Das sogenannte Positionspapier zur Novellierung der Approbationsordnung hält auch an diesen Gebieten weiter fest, wobei die instrumentelle Analytik im Vergleich zur klassischen stärker betont wird und die pharmazeutische Biologie ein stärkeres Gewicht auf die Grundlagen der Immunologie und der Impfstoffe legt. Die gerade für die pharmazeutische Technologie wichtigen Grundlagenfächer physikalische Chemie, Physik und Mathematik werden als physikalische Pharmazie zusammengefasst.

Im Positionspapier werden Pharmakologie und klinische Pharmazie neu in das Grundstudium aufgenommen. In diese Fächer werden auch die Grundlagen der Humanbiologie integriert. Das Positionspapier ist vom sogenannten runden Tisch erarbeitet worden. Mitglieder waren Vertreter aller pharmazeutischen Interessengruppen (siehe auch den Beitrag von Winter in diesem Heft).

## Abstract (en)

Pharmacy is part of the natural sciences and therefore the teachers in pharmacy provide basic training based on these subjects, although unlike the other natural science subjects, pharmacy is regulated by a standardised federal licensing regulation. As expected, general, organic and physical chemistry; analytics; biology; biochemistry; human biology; mathematics and physics in particular are included as basic subjects. The so-called “Position Paper on the Reorganisation of the Licensing Regulations” also continues to adhere to these areas, whereby instrumental analysis is emphasised more strongly in comparison to classical analysis and pharmaceutical biology places greater emphasis on the fundamentals of immunology and vaccines. Physical chemistry, physics and mathematics, which are particularly important for pharmaceutical technology, are integrated in physical pharmacy.

In the position paper, pharmacology and clinical pharmacy are newly included in the basic study programme. The basics of human biology are also integrated into these subjects.

The position paper is the basis of the so-called “Round Table”. Members of the Round Table are members of all interest groups related to the German pharmacy (see also the article of Winter in this volume).

## Einleitung

Im Gegensatz zu allen anderen Studienfächern sind Heilberufe wie die Pharmazie durch bundeseinheitliche Approbationsordnungen geregelt und unterliegen von daher keiner weiteren Akkreditierung. Dies schränkt die einzelnen Standorte und deren Dozenten bezüglich der Inhalte ihrer Lehrveranstaltungen stark ein, obwohl laut Grundgesetz diese die Freiheit von Forschung und Lehre genießen [1]. Dies kann mit dem Hinweis auf einheitliche Standards bei Heilberufen gerechtfertigt werden. Natürlich kann ein Pharmaziedozent aus seiner Sicht wichtige Inhalte vermitteln, auch wenn diese nicht Teil des Gegenstandskatalogs sind. In der Praxis führt dies aber dazu, im Interesse der Studierenden bei den Schwerpunkten sich am Gegenstandskatalog zu orientieren. Insofern kann die Aufnahme der naturwissenschaftlichen Grundlagen in die Apothekerausbildung auf der Basis der gültigen Approbationsordnung und deren hoffentlich bald anstehender Novellierung und daraus resultierender Gegenstandskataloge diskutiert werden.

Die Zielsetzung dieser Arbeit besteht nicht darin, Vor- und Nachteile der bestehenden Approbationsordnung im Vergleich zu der vorgeschlagenen Novellierung in der gesamten Breite zu diskutieren, sondern der Beitrag bezieht sich nur auf Bewertung und Fragestellungen bezüglich der naturwissenschaftlichen Grundlagen der Pharmazeutenausbildung.

## Einordnung der Pharmazie in den Fächerkanon der universitären Fächer

Die Ausbildung der Pharmazeuten, die auch zur Approbation zum Apotheker führt, ist Bestandteil der naturwissenschaftlichen Fächer. Von daher sind die pharmazeutischen Institute in Deutschland mit nur wenigen Ausnahmen mit anderen naturwissenschaftlichen Fächern zu Fachbereichen oder mathematisch-naturwissenschaftlichen Fakultäten vereint. Organisatorisch ist die Pharmazie neben der Biologie, Chemie, Physik und Mathematik ein Fach des Mathematisch-Naturwissenschaftlichen Fakultätentages (MNFT) und übernimmt seit einiger Zeit turnusmäßig auch das Sprecheramt. Die Mitgliedschaft im MNFT unterstreicht die Verankerung der Pharmazie in den Naturwissenschaften. Über den MNFT ist die Pharmazie auch im Allgemeinen Fakultätentag (AFT) vertreten.

Pharmazie stellt darüber hinaus aber auch die Verbindung zu den medizinischen Fakultäten dar, welches sich nicht nur in vielen interdisziplinären Forschungsaktivitäten, sondern auch in gemeinsamen Lehrveranstaltungen, wie z. B. die der Pharmakologie, niederschlägt. Dieser duale Charakter ist wahrscheinlich die Ursache dafür, dass die Inhalte des Pharmaziestudiums wie kaum in einem anderen Fach so stark in den eigenen Reihen diskutiert werden. Höhepunkte waren die intensiven kontroversen Auseinandersetzungen bei der Novellierung der Approbationsordnung von 2001.

## Regelungen der gültigen Approbationsordnung zur Grundausbildung der Apotheker

Die Ausbildung der Pharmazeuten ist in 3 Abschnitte unterteilt. Das Grundstudium wird auch als „Erster Abschnitt“ bezeichnet und beträgt 4 Semester. Daran schließt sich das Erste Staatsexamen an. Die darauf aufbauenden weiteren 4 Semester nennt man den „Zweiten Abschnitt“, der mit dem „Zweiten Staatsexamen“ endet. Der „Dritte Abschnitt“ der Pharmazeutenausbildung beinhaltet das sogenannte Praktische Jahr, welches ein halbes Jahr in einer öffentlichen Apotheke absolviert werden muss. Das zweite halbe Jahr kann wahlweise wieder in der öffentlichen Apotheke, aber auch in der Industrie, Krankenhauspharmazie, Bundeswehr und in vielen anderen Einrichtungen mit pharmazeutischen Fragestellungen absolviert werden. An das Ende der Ausbildung schließt sich das „Dritte Staatsexamen“ an, das Bestehen der Prüfung ist die Voraussetzung zur Beantragung der Approbation.

Die bundeseinheitliche Approbationsordnung [2] legt in § 17 4 Fächer für den ersten Prüfungsabschnitt (Erstes Staatsexamen) und damit die Grundlagen der Ausbildung fest. In der Anlage 13 zu § 17 finden sich Gegenstandskataloge zu den einzelnen Prüfungsfächern (Tab. 1).

**Tab. 1:** Erster Abschnitt der Pharmazeutischen Prüfung (Anlage 13 zu § 17 Abs. 3 der gültigen Approbationsordnung für Apotheker; [2])

I	Allgemeine, anorganische und organische Chemie
	Grundbegriffe und -gesetze der Chemie; Atombau und Periodensystem der Elemente; chemische Bindung, zwischenmolekulare Bindungskräfte, Lösungen und heterogene Systeme; Thermodynamik chemischer Reaktionen sowie Reaktionskinetik; chemisches Gleichgewicht; Säure/Base- und Redoxsysteme; Stöchiometrie chemischer Reaktionen
	Vorkommen, Gewinnung, Eigenschaften und Reaktivität von Elementen des Periodensystems und ihrer Verbindungen sowie deren Herstellung; allgemeine Chemie der Arzneistoffe, Hilfsstoffe und Schadstoffe; Summenformeln und Geometrie wichtiger Verbindungen; Nomenklatur
	Bindungsarten und ihre theoretischen Grundlagen; Reaktionsgleichungen und -mechanismen; Grundlagen der Stereochemie; Chemie funktioneller Gruppen und Stoffklassen sowie ihre Herstellung und Eigenschaften; Summen‑, Struktur- und Stereoformeln; Eigenschaften und Reaktivität von Synthetika und Naturstoffen; chemische Grundlagen von synthetischen Polymeren und Biopolymeren; Nomenklatur
II	Grundlagen der pharmazeutischen Biologie und der Humanbiologie
	Grundlagen der Zytologie und Histologie; Grundprinzipien und molekulare Grundlagen des Stoffwechsels und der Genetik; Merkmale, systematische Einteilung und Physiologie von Pflanzen und Mikroorganismen unter besonderer Berücksichtigung pharmazeutisch und medizinisch wichtiger Organismen; Viren
	Grundlagen der Anatomie und Morphologie von Pflanzen; ökologische Grundbegriffe; drogenkundliche und mikrobiologische Grundbegriffe und Techniken; wichtige Arznei- und Giftpflanzen; Stammpflanzen gebräuchlicher Drogen
	Makroskopischer und mikroskopischer Aufbau des menschlichen Körpers, seine Organe und Gewebe; Funktion von Organen und Organsystemen unter Einschluss von Regulationsmechanismen und zellbiologischen Aspekten; Grundzüge des Immunsystems; Fortpflanzungsorgane und deren Funktion, Schwangerschaft; Zusammensetzung und Umfang normaler Ernährung
III	Grundlagen der Physik, der physikalischen Chemie und der Arzneiformenlehre
	Grundbegriffe und Maßsysteme der Physik; Grundgesetze der Mechanik fester Körper, Flüssigkeiten und Gase; Aggregatzustände und deren Änderungen; Phasensysteme; Grenzflächenerscheinungen; Grundlagen der Thermodynamik und -kinetik; Kinetik der Diffusion und Verteilung; Grundlagen der Elektrizitätslehre, einschließlich Elektrochemie; Grundlagen der Optik, Schwingungs- und Wellenlehre; Aufbau und Eigenschaften der Atome und Moleküle; Grundlagen der Radioaktivität und Isotopenanwendung; physikalische Grundlagen von Messverfahren jeweils unter Berücksichtigung der Belange der Pharmazie
	Allgemeine Anforderungen an die Herstellung von Arzneimitteln; Grundoperationen; Rezepturarzneimittel; homöopathische Zubereitungen
IV	Grundlagen der pharmazeutischen Analytik
	Die in der pharmazeutischen Analytik gebräuchlichen, grundlegenden Methoden; Grundlage, Arbeitsweisen und Anwendung klassischer qualitativer und quantitativer Verfahren zur Analyse von Arzneistoffen, Hilfsstoffen und Schadstoffen (Kationen, Anionen und Neutralstoffen), einschließlich der Arzneibuch-Methoden; Analytik funktioneller Gruppen organischer Verbindungen
	Instrumentelle pharmazeutische Analyseverfahren, einschließlich spurenanalytischer Verfahren: Grundlagen, Arbeitsweisen und Anwendungen elektrochemischer, thermoanalytischer, radiochemischer, chromatografischer, optischer und spektroskopischer Verfahren zur qualitativen (Identifizierung und Strukturaufklärung) und quantitativen Analyse; Validierung von Analyseverfahren; Qualitätssicherung

Seit 1976 ist das Institut für Medizinische und Pharmazeutische Prüfungsfragen (IMPP) in Mainz zuständig für die Durchführung des Ersten Staatsexamens und damit für die Grundlagen der Ausbildung zum Apotheker [3]. Hierzu ist auf der Basis der Approbationsordnung ein Gegenstandskatalog [4] erstellt worden, der zwar recht ausführlich ist, aber auch offenlässt, in welcher Tiefe die einzelnen Gebiete behandelt werden sollen. Das IMPP arbeitet zurzeit daran, diesen Gegenstandskatalog in einen kompetenzorientierten Katalog umzuwandeln.

Die Universitätsausbildung zur Vermittlung des Wissens und der Kompetenzen sind in der Anlage 1 zu § 2 Abs. 2 der Approbationsordnung in Form von Stoffgebieten beschrieben, wobei sich A–D auf die Grundausbildung beziehen. Diese Stoffgebiete sind in der Tab. 2 wiedergegeben [2]. Auch wenn dieser Musterstudiengang keinen Gesetzescharakter hat, ist von der Kommission zur Novellierung der Approbationsordnung im Jahr 2001 ein solcher erstellt worden [5]. Dieser findet sich auch auf der Homepage der „Konferenz der Fachbereiche Pharmazie“ (KFPharm; [6]). Mitglieder der Kommission waren Vertreter des Bundesministeriums der Gesundheit, der Professoren aus der Pharmazie, des pharmazeutischen, akademischen Mittelbaus, der Pharmaziestudierenden, der Pharmaindustrie, der Krankenhauspharmazie und der Landesministerien. Beim Musterstudiengang ist eine Unterteilung der einzelnen Lehrveranstaltungen zu den Kernfächern der Ausbildung pharmazeutische/medizinische Chemie, pharmazeutische Biologie, pharmazeutische Technologie, Pharmakologie/klinische Pharmazie und Geschichte der Pharmazie vorgenommen worden, wobei diese keine festen Regelungen darüber trifft, welches Fach die jeweilige Lehrveranstaltung anbietet. Sie ermöglicht es den einzelnen Standorten je nach Ausrichtung der Forschungseinrichtungen der einzelnen Dozenten eine Zuteilung vorzunehmen. Auch die Pharmazie sollte an dem klassischen Konzept der Verknüpfung von Lehre und Forschung an einer Universität so weit wie möglich festhalten.

**Tab. 2:** Stoffgebiete des Studiums der Pharmazie (Auszug aus Anlage 1 zu § 2 Abs. 2 der gültigen Approbationsordnung für Apotheker; [2])

*Stoffgebiet A*
Allgemeine Chemie der Arzneistoffe, Hilfsstoffe und Schadstoffe
Vorlesungen, Seminare und praktische Übungen mit Veranstaltungen zu:
Chemie für Pharmazeuten
Stereochemie
Chemische Nomenklatur
Allgemeine und analytische Chemie der anorganischen Arzneistoffe, Hilfsstoffe und Schadstoffe (unter Einbeziehung von Arzneibuch-Methoden)
Chemie, einschließlich der Analytik der organischen Arzneistoffe, Hilfsstoffe und Schadstoffe
Toxikologie der Hilfsstoffe und Schadstoffe
Gesamtumfang: 462 Unterrichtsstunden mit einem Anteil von 336 Unterrichtsstunden praktische Übungen und 56 Unterrichtsstunden Seminare
Drei Bescheinigungen über die erfolgreiche und regelmäßige Teilnahme
*Stoffgebiet B*
Pharmazeutische Analytik
Vorlesungen, Seminare und praktische Übungen mit Veranstaltungen zu:
Pharmazeutische/Medizinische Chemie
Quantitative Bestimmung von Arznei‑, Hilfs- und Schadstoffen (unter Einbeziehung von Arzneibuch-Methoden)
Einführung in die instrumentelle Analytik
Instrumentelle Analytik
Gesamtumfang: 392 Unterrichtsstunden mit einem Anteil von 308 Unterrichtsstunden praktische Übungen
Zwei Bescheinigungen über die erfolgreiche und regelmäßige Teilnahme
*Stoffgebiet C*
Wissenschaftliche Grundlagen, Mathematik und Arzneiformenlehre
Vorlesungen, Seminare und praktische Übungen mit Veranstaltungen zu:
Physik für Pharmazeuten
Grundlagen der physikalischen Chemie
Physikalische Übungen für Pharmazeuten
Physikalisch-chemische Übungen für Pharmazeuten
Mathematische und statistische Methoden für Pharmazeuten
Grundlagen der Arzneiformenlehre
Arzneiformenlehre
Pharmazeutische und medizinische Terminologie
Geschichte der Naturwissenschaften unter besonderer Berücksichtigung der Pharmazie
Gesamtumfang: 280 Unterrichtsstunden mit einem Anteil von 140 Unterrichtsstunden praktische Übungen und 14 Unterrichtsstunden Seminare
Drei Bescheinigungen über die erfolgreiche und regelmäßige Teilnahme
*Stoffgebiet D*
Grundlagen der Biologie und Humanbiologie
Vorlesungen, Seminare und praktische Übungen mit Veranstaltungen zu:
Allgemeine Biologie für Pharmazeuten
Systematische Einteilung und Physiologie der pathogenen und arzneistoffproduzierenden Organismen
Pharmazeutische Biologie I (Untersuchungen arzneistoffproduzierender Organismen)
Arzneipflanzenexkursionen, Bestimmungsübungen
Mikrobiologie
Pharmazeutische Biologie II (pflanzliche Drogen)
Zytologische und histologische Grundlagen der Biologie
Grundlagen der Anatomie und Physiologie
Kursus der Physiologie
Grundlagen der Biochemie
Grundlagen der Ernährungslehre
Gesamtumfang: 392 Unterrichtsstunden mit einem Anteil von 210 Unterrichtsstunden praktische Übungen
Vier Bescheinigungen über die erfolgreiche und regelmäßige Teilnahme

Kommissionen der „Konferenz der Fachbereiche Pharmazie“, damals noch „Verband der Pharmazieprofessoren“, haben hieraus entsprechende Qualifikationsziele erstellt, die auf der Homepage innerhalb der Modulbeschreibungen zu finden sind [7]. Die Beschreibungen erfolgten auf Modulebene, da zu dem Zeitpunkt im Rahmen der Umstellung auf Bachelor‑/Masterstudiengänge darum gebeten worden war. Wie bekannt, ist die Pharmazie dann aber doch nicht zu diesem System gewechselt.

Es liegen also umfangreiche Gesetze und Materialien vor, die den Istzustand der grundlegenden Ausbildung zum Apotheker festlegen bzw. beschreiben. Mit 4 Semestern Grundausbildung zeigt sich die gesamte Breite des Pharmaziestudiums. Auf therapiebezogene Ansätze wird dabei weitgehend verzichtet. Dies ist nicht ohne Kritik geblieben, sodass im Positionspapier des sogenannten runden Tisches Grundlagen der Fächer klinische Pharmazie und Pharmakologie in den Ersten Abschnitt einbezogen worden sind [8], um den Studierenden schon von Anfang an einen Blick auf den Patienten werfen zu lassen (siehe zum Positionspapier auch den Beitrag von Winter in diesem Themenheft).

## Weiterentwicklung der naturwissenschaftlichen Fächer im Grundstudium

Im Folgenden soll die Weiterentwicklung der Approbationsordnung fächerbezogen beschrieben und diskutiert werden, wobei die Vorschläge aus dem Positionspapier einfließen. Zum Vergleich werden in Tab. 3 Auszüge aus dem Musterstudiengang und in Tab. 4 die entsprechenden Angaben aus dem Positionspapier dargestellt. Das Positionspapier beschreibt keine Stoffgebiete, sondern einzelne Module, bei denen Praktikum, Seminar und Vorlesung den gleichen Titel tragen. Der Umfang in Semesterwochenstunden (SWS) wurde aufgenommen. Die 4 Fächer des Ersten Staatsexamens laut § 17 der gültigen Approbationsordnung werden der Darstellung zugrunde gelegt (Tab. 1).

**Tab. 3:** Auszug aus dem Musterstudiengang der novellierten Approbationsordnung von 2001, bezogen auf die naturwissenschaftliche Ausbildung im Grundstudium [5]

Titel der Veranstaltungen	Veranstaltungsart und -umfang (SWS)
*Fächergruppe I*	
Chemie für Pharmazeuten	V 70
Einführung in die instrumentelle Analytik	V 42
Pharmazeutische/Medizinische Chemie	V 42
Stereochemie	S 14
Chemische Nomenklatur	S 14
Allgemeine und analytische Chemie der anorganischen Arznei‑, Hilfs- und Schadstoffe (unter Einbeziehung von Arzneibuch-Methoden)	P 168
Quantitative Bestimmung von Arznei‑, Hilfs- und Schadstoffen (unter Einbeziehung von Arzneibuch-Methoden)	P 140
Instrumentelle Analytik	P 168
Chemie der organischen Arznei‑, Hilfs- und Schadstoffe	P 168
*Fächergruppe II*	
Allgemeine Biologie für Pharmazeuten, systematische Einteilung und Physiologie der pathogenen und arzneistoffproduzierenden Organismen	V 70
Pharmazeutische Biologie I (Untersuchungen arzneistoffproduzierender Organismen)	P 42
Arzneipflanzenexkursionen, Bestimmungsübungen	P 28
Mikrobiologie	P 42
Grundlagen der Biochemie	V 14
Pharmazeutische Biologie II (pflanzliche Drogen)	P 42
*Fächergruppe III*	
Physik für Pharmazeuten	V 42
Grundlagen der physikalischen Chemie	V 28
Grundlagen der Arzneiformenlehre	V 28
Mathematische und statistische Methoden für Pharmazeuten	V + Ü 28
Physikalische Übungen für Pharmazeuten	P 28
Physikalisch-chemische Übungen für Pharmazeuten	P 28
Arzneiformenlehre	P 70
*Fächergruppe IV*	
Pharmazeutische und medizinische Terminologie	S 14
Zytologische und histologische Grundlagen der Biologie	P 28
Grundlagen der Anatomie und Physiologie	V 84
Toxikologie der Hilfs- und Schadstoffe	S 28
Grundlagen der Ernährungslehre	V 14
Kursus der Physiologie	P 28

*V* Vorlesung; *P* Praktikum; *S* Seminar; *SWS* Semesterwochenstunden

**Tab. 4:** Positionspapier „runder Tisch“: „Novellierung der Approbationsordnung“, bezogen auf die naturwissenschaftliche Ausbildung im Grundstudium [8]

Modul		Praktikum	Vorlesung	Seminar	Insgesamt
*Module der pharmazeutischen/medizinischen Chemie*					
1	Chemische und analytische Grundkompetenzen in den pharmazeutischen Wissenschaften *Teil 1: Chemische Grundlagen und analytische Grundkompetenzen*	12	5	–	17
2	Chemische und analytische Grundkompetenzen in den pharmazeutischen Wissenschaften *Teil 2: Organische Chemie der Arznei‑, Hilfs- und Schadstoffe*	10	4	–	14
	Seminar Nomenklatur	–	–	1	1
	Seminar Stereochemie	–	–	1	1
3	Chemische und analytische Grundkompetenzen in den pharmazeutischen Wissenschaften *Teil 3: Instrumentelle Analytik*	12	4	–	16
4	Chemische und analytische Grundkompetenzen in den pharmazeutischen Wissenschaften *Teil 4: Grundlagen der Biochemie*	4	2	–	6
*Module der pharmazeutischen Biologie*					
1	Grundlagen der Biologie und Biochemie	3	5	–	8
2	Medizinische und pharmazeutische Mikrobiologie	2	2	–	4
3	Grundlagen der Phytopharmazie	3	2	–	5
4	Grundlagen der Immunologie und Impfstoffe	–	4	–	4
*Module der pharmazeutischen Technologie*					
1	Grundlagen und Messverfahren der physikalischen Pharmazie	3	2	–	5
2	Pharmazeutisch-technologische Hilfsstoffe und Grundoperationen	3	2	–	5
*Module der Pharmakologie und Toxikologie*					
1	Grundlagen der Anatomie, Histologie, Physiologie und Ernährungslehre	4	4	2	10
2	Grundlagen der Pharmakologie und Toxikologie	2	2	2	6
*Module der klinischen Pharmazie*					
1	Grundlagen der Anatomie, Histologie, Physiologie und Ernährungslehre	–	–	1	1
2	Grundlagen der klinischen Pharmazie	–	2	3	5
*Module der Geschichte der Pharmazie*					
1	Theoretische, historische und kommunikative Grundlagen				
	*Teil a) Pharmazeutische und medizinische Terminologie*	–	1	1	2
	*Teil b) Geschichte und Theorie der Naturwissenschaften unter besonderer Berücksichtigung der Pharmazie*	–	2	–	2

Angaben in Semesterwochenstunden *SWS*

### Allgemeine, anorganische und organische Chemie

Der Chemie kommt als Grundlagenwissenschaft besondere Bedeutung zu, da auch alle anderen Teilfächer zum großen Teil darauf aufbauen. Chemische Strukturen bedingen sowohl die Pharmakodynamik als auch die Pharmakokinetik von Arznei- und Wirkstoffen. Sie stellen auch die Grundlagen für Interaktionen und Instabilitäten dar. Deswegen ist eine umfassende chemische Grundlagenausbildung für eine strukturbasierte Pharmazie unabdingbar. Insofern überrascht es nicht, dass die Pharmazie aus der Chemie und Medizin hervorgegangen ist. In der lange gültigen akademischen Ausbildungsordnung der Pharmazeuten von 1934 dominierte die Chemie mit zahlreichen Praktika von ca. 23 SWS. Unter Chemie ist allerdings auch die pharmazeutische Analytik (siehe unter d) zu verstehen. Aber das organische Praktikum nahm ebenfalls einen großen Raum mit der Synthese zahlreicher Präparate ein.

Der Anteil der Chemie ist im Laufe der Approbationsordnungen, vor allem der zugehörigen praktischen Lehrveranstaltungen, zurückgefahren worden (Details zur Entwicklung der akademischen Apothekerausbildung auch in der DDR finden sich in [9]). Den stärksten Einbruch erlebte die Chemie bei der Novellierung der Approbationsordnung von 1989 im Jahr 2001. Die pharmazeutische/medizinische Chemie und damit auch die Grundausbildung wurden insbesondere auf Kosten der Fächer Pharmakologie und klinische Pharmazie stark gekürzt. Bei der Betrachtung der Praktika sind auch die zugehörigen Seminarstunden zu bedenken, die zwar nicht eindeutig vorgeschrieben waren, aber bei der jetzt gültigen Approbationsordnung mit 20 % festgelegt sind. Dies sollte auch bei einer neuen Approbationsordnung so übernommen werden.

Die Lehrveranstaltungen im Ersten Abschnitt, die den Pharmazeuten angeboten werden, sind immer wieder Gegenstand von Diskussionen. Diese beziehen sich darauf, ob diese Grundlagen von den Pharmazeuten selber oder von anderen Naturwissenschaftlern vermittelt werden sollen. So wünschen sich viele die Teilnahme der Pharmaziestudierenden an den grundlegenden Vorlesungen, die von Dozenten der Chemie gehalten werden. An diesen Vorlesungen für Chemiker und Biologen teilzunehmen, bietet den Vorteil, dass Pharmaziestudierende auch über den Tellerrand der Pharmazie hinausschauen und die Zugehörigkeit zu den naturwissenschaftlichen Fächern gefördert wird. Die meisten Institute sind allerdings immer mehr dazu übergegangen, eigenständige Vorlesungen anzubieten. Hier wird der Vorteil gesehen, dass die Lehrveranstaltungen an die Bedürfnisse von Pharmazeuten angepasst und optimiert werden können. Insofern besteht bei einigen der Wunsch, derartige Lehrveranstaltungen möglichst umfänglich innerhalb der Pharmazie anzubieten.

Wenn eben machbar, sollte hier beides ermöglicht werden: Eine große Breite an Experimentalvorlesungen für alle Studierenden der Naturwissenschaften und spezielle Angebote für Studierende der Pharmazie. Wie in allen anderen grundlegenden Vorlesungen stellt sich auch bei diesen chemieorientierten Veranstaltungen die Frage, in welcher Tiefe man einzelne Teilgebiete bearbeiten soll. Hier muss immer der Blick auf die Anforderungen des Zweiten Abschnittes des Studiums gerichtet sein. Die ausführliche Darstellung der Komplexchemie stellt beispielsweise eine notwendige Voraussetzung zum tiefergehenden Verständnis der Wechselwirkungen von Arzneistoffen dar.

An diesem Beispiel wird auch deutlich, dass Biochemie für die Pharmazeuten immer wichtiger geworden ist. Nicht nur zum Verständnis des Metabolismus, sondern generell zum Blick auf die Beeinflussung von Biomakromolekülen durch Arzneistoffe. Ein größerer Umfang im Vergleich zur bestehenden Approbationsordnung ist sinnvoll. Insofern ist es zu begrüßen, dass im Positionspapier sowohl in der pharmazeutischen/medizinischen Chemie als auch in der pharmazeutischen Biologie entsprechende Module aufgenommen worden sind. In der Summe ist die Anzahl der Praktikumsstunden allerdings im Vergleich zur bestehenden Approbationsordnung gleichgeblieben. Wer die biochemischen Lehrveranstaltungen anbietet, sollte vor Ort anhand der jeweiligen Forschungsrichtungen der einzelnen Dozenten entschieden werden.

Bei der anorganischen Chemie können in Bezug auf die reine „Stoffchemie“ durchaus Abstriche gemacht werden. Dieses Gebiet sollte auch entsprechend im Ersten Abschnitt weiter zurückgefahren werden. Man könnte sich hierbei auf die vorliegenden anorganischen Arzneistoffe konzentrieren.

Auf der anderen Seite ist die organische Chemie zum Verständnis der Wirkung auf molekularer Ebene unabdingbar. Während die Synthese der Arzneistoffe bei früheren Ausbildungen absolut im Vordergrund stand, hat dieses Fach bei den jüngeren Approbationsordnungen an Bedeutung verloren. Es sollte auf keinen Fall auf ein organisches Praktikum mit grundlegenden Synthesen und zugehörigen Lehrveranstaltungen verzichtet werden. Wünschenswert ist dabei natürlich, soweit wie möglich auf einfache Arzneistoffe zurückzugreifen. Im Positionspapier ist von daher im Grundstudium wieder ein entsprechendes Praktikum vorgesehen, das durch das Modul „Synthetische Arzneistoffe“ im Zweiten Abschnitt vertieft wird.

### Grundlagen der pharmazeutischen Biologie und der Humanbiologie

Das Fach pharmazeutische Biologie ist zum Verständnis der Arzneistoffe biogenen Ursprungs für die Ausbildung der Pharmazeuten in Deutschland ebenfalls unabdingbar. Die Ausbildung der pharmazeutischen Biologie soll nicht nur die Grundlagen der Biologie, sondern auch die der Biochemie (neben der pharmazeutischen/medizinischen Chemie) und der Gentechnik einschließen, um die Voraussetzungen für das Verständnis der vielen neuen Biologika und Biopharmazeutika zu schaffen, welche dann im Hauptstudium im Detail besprochen werden können.

Viele der oben gemachten Anmerkungen zur allgemeinen, anorganischen und organischen Chemie gelten auch für die pharmazeutische Biologie und Humanbiologie, wie zum Beispiel zur Bedeutung der Biochemie. Die Grundvorlesungen und Praktika der pharmazeutischen Biologie werden überwiegend von deren Dozenten abgehalten. Es werden weniger Diskussionen darüber geführt, ob man hier gemeinsame Veranstaltungen mit der Biologie abhalten sollte, wie es bei der Approbationsordnung aus dem Jahr 1934 noch der Fall war. Die klassische pharmazeutische Biologie soll laut Positionspapier neben der Besprechung der Phytopharmazie und Phytotherapeutika zwar etwas zurückgefahren werden, aber weiter wichtiger Bestandteil der Ausbildung bleiben.

Aufgrund der hohen Bedeutung von Impfstoffen muss die Vorlesung „Grundlagen der Immunologie und Impfstoffe“ auf die Immunpathologie und die Immuntherapeutika im Zweiten Abschnitt vorbereiten. Insofern kann man auch hier die entsprechenden grundlegenden Veranstaltungen des Ersten Abschnittes im Positionspapier nur begrüßen.

Die Humanbiologie als zweiter Teil dieses Prüfungsfaches lehnt sich an die medizinische Ausbildung an und bildet mit der Betrachtung des makroskopischen und mikroskopischen Aufbaus des menschlichen Körpers, seiner Organe, Gewebe und Funktionen die absolut notwendige Grundlage zum Verständnis der Anwendung von Arzneistoffen im Zweiten Abschnitt. Die entsprechenden Module finden sich im Positionspapier jetzt hauptsächlich im Grundstudium der Pharmakologie und Toxikologie mit 10 SWS und 1 SWS bei der klinischen Pharmazie, wie dies bisher auch der Fächergruppe IV (Tab. 3) zugeordnet worden war. Dies erscheint sinnvoll.

### Grundlagen der Physik, der physikalischen Chemie und der Arzneiformenlehre

In den Lernzielen und Inhalten der Praktika und Vorlesungen innerhalb der Modulbeschreibungen [10] hat die Konferenz der Fachbereiche Pharmazie festgehalten: „Der Bereich Pharmazeutische Technologie und Biopharmazie, der sich im weitesten Sinne mit der Herstellung von Arzneiformen befasst, ist ein Kernbestandteil der pharmazeutischen Ausbildung. Im Basisstudium werden innerhalb der Lehrveranstaltungen in diesem Bereich zunächst die theoretischen Grundlagen in den Fächern Mathematik, Physik und Physikalische Chemie gelegt und dann im Bereich pharmazeutische Technologie anwendungsbezogen vertieft. Stufenweise werden in den Vorlesungen und Seminaren die Herstellung von Arzneimitteln, die Qualitätsanforderungen und die arzneiformenbezogenen biopharmazeutischen Eigenschaften erarbeitet.“ Diese Zielsetzung soll in der Form weiter verfolgt werden, wobei allerdings die Vorlesungen „Physik für Pharmazeuten“ und „Grundlagen der Physikalischen Chemie“ mit den zugehörigen Praktika sowie die „Mathematischen und Statistischen Methoden für Pharmazeuten“ (siehe Fächergruppe III in Tab. 3) nicht mehr auftauchen. „Physik“ und „Physikalische Chemie“ sind bei Modul I durch den Begriff „Physikalische Pharmazie“ ersetzt worden. Dadurch wird ein stärkerer Bezug zur Pharmazie hergestellt und die für die Pharmazie notwendigen Grundlagenfächer physikalische Chemie, Mathematik und Physik werden zusammengefasst. Hier schlägt sich erneut die Diskussion nieder, ob die Grundlagen von Dozenten aus der Pharmazie oder anderen Naturwissenschaften vermittelt werden sollen. Da die Thematik der Arzneiformenlehre sehr nah an der Pharmazie und weniger an anderen Gebieten liegt, kann man sich auf den Standpunkt stellen, auch diese Grundlagen eher in die Pharmazie zu verlegen. Allerdings sind einige dieser Lehrveranstaltungen auch schon in der Vergangenheit von Professoren der pharmazeutischen Technologie übernommen worden.

Im zweiten Modul will man sich auf „Pharmazeutisch-Technologische Hilfsstoffe und Grundoperationen“ konzentrieren. Dies rückt an die Stelle der „Arzneiformenlehre“ im Grundstudium der bestehenden Approbationsordnung. Bisher wurde die individualisierte Arzneimittelherstellung, also auch die in der öffentlichen Apotheke, im Grundstudium vermittelt. Jetzt soll die Herstellung vollständig in das Hauptstudium verschoben werden. Trotzdem soll mit den neuen Modulen ein stärkerer und zeitlich früherer Praxisbezug geschaffen werden. Grundoperationen bedeutet zum Beispiel, dass hier bereits Herstellungsgrundlagen geschaffen werden und Herstellungsprozesse beleuchtet werden.

### Grundlagen der pharmazeutischen Analytik

Analytik ist im Bereich der Pharmazie immer dominanter geworden. Gerade in der Forschung profitieren alle von modernen analytischen Verfahren. Allerdings beschränkte man sich sehr lange Zeit in der Ausbildung der Pharmazeuten auf die klassische Analytik, sodass auch in der jetzt gültigen Approbationsordnung 2 größere Praktika zur „Qualitativen Analytik“ und „Quantitativen Analytik“ vorgesehen sind. Mit Einführung der Approbationsordnungen von 1971 und 1989 gewann die „Instrumentelle Analytik“ immer mehr an Bedeutung. Da im Bereich der Forschung die klassische Analytik so gut wie verschwunden ist, könnte man hier stark streichen. Allerdings müssen auch für die öffentliche Apotheke entsprechende apothekengerechte Methoden vorgesehen sein, die auch noch in den Arzneibüchern auftauchen. Insofern kann man diese Grundpraktika nicht vollends aus der Ausbildung herausnehmen, zumal man an diesen Verfahren auch sehr gut allgemeine Chemie erklären kann. Der Kompromiss, die bisherigen Praktika der qualitativen und der quantitativen Analytik im Modul I zusammenzuziehen und auch hier die chemischen Grundlagen mit aufzunehmen, ist nachvollziehbar, zumal die instrumentelle Analytik im Grundstudium nicht weiter gekürzt worden ist. Beim Modul III in den Grundkompetenzen müssen dabei auch die Grundlagen für Modul V, in dem Arzneistoff- und Arzneimittelanalytik vereint worden sind, und für Modul VI im Hauptstudium vermittelt werden, welches mit Bioanalytik, Drug Monitoring, diagnostischer und klinischer Chemie umschrieben wird. Dieses Modul im Hauptstudium stellt sicherlich einen großen Fortschritt im Vergleich zur bestehenden Approbationsordnung dar. Vielleicht hätte man hier im Grundstudium vom Modul III noch etwas zulegen können.

## Fazit

Chemie und Biologie mit allen Teildisziplinen stellen mit Recht nach wie vor die wichtigsten Grundlagen für die Ausbildung der Pharmazeuten dar, wobei die Kombination aus beiden Fächern, die Biochemie, stärker an Bedeutung gewinnt. Verschiebungen im Bereich der pharmazeutischen Biologie zugunsten der Gentechnik und Immunologie sind ebenfalls begrüßenswert. Dies trifft auch auf die Stärkung der „Instrumentellen Analytik“ im Vergleich zur „Klassischen Analytik“ zu. Die pharmazeutische Technologie führt die Vermittlung von Grundlagen in dem Fach Physik/klinische Pharmazie zusammen.

Ein Vergleich der SWS zwischen Positionspapier und gültiger Approbationsordnung im Grundstudium ist dadurch erschwert, dass einige „Umverteilungen“ zwischen den Fächern, aber auch zwischen Haupt- und Grundstudium vorgenommen worden sind.

Insgesamt will man an den Grundlagen der naturwissenschaftlichen Grundausbildung der Pharmazeuten nicht rütteln, was sicherlich auch deren Stellung innerhalb der Naturwissenschaften gefährden würde. Dem Festhalten an einer einheitlichen Approbation zur Ausbildung in sämtlichen Bereichen der Pharmazie wird auch dadurch Rechnung getragen, dass man die Grundlagen zur Forschung weiter beibehalten will. Die gewünschten Zuwächse an mehr patientenorientierter Pharmazie sind ebenfalls konsensfähig, können aber nur durch Verlängerung des Studiums erzielt werden. Dies ist der Kernpunkt des Positionspapiers und man kann nur auf eine rasche Umsetzung durch das BMG (Bundesministerium der Gesundheit) hoffen.
